# The HUPO Human Proteome Project (HPP), a Global Health Research Collaboration

**DOI:** 10.5195/cajgh.2012.37

**Published:** 2012-09-20

**Authors:** Gilbert S. Omenn

**Affiliations:** Director, Center for Computational Medicine and Bioinformatics; Professor of Internal Medicine, Human Genetics, and Public Health, University of Michigan; Chair, global Human Proteome Project

**Keywords:** Human Proteome Project, proteome

## Abstract

The global Human Proteome Project (HPP) was announced by the Human Proteome Organization (HUPO) at the 2010 World Congress of Proteomics in Sydney, Australia, and launched at the 2011 World Congress of Proteomics in Geneva, Switzerland, with analogies to the highly successful Human Genome Project. Extensive progress was reported at the September 2012 World Congress in Boston, USA. The HPP is designed to map the entire human proteome using available and emerging technologies.

The HPP aims to create a molecular and biological foundation for improving health globally through better understanding of disease processes, more accurate diagnoses, and targets for more effective therapies and preventive interventions against many diseases. There are opportunities for individual investigators everywhere to access advanced datasets and to join HPP research teams.

## Introduction

As described at BioVision 2012 at the Bibliotheca Alexandrina in Alexandria, Egypt, in April 2012, the Human Genome Project has dramatically increased knowledge of inherited diseases and the genetic contribution to all kinds of complex diseases.^1^ The biological and now clinical progress in genomics made feasible remarkable advances in technologies for sequencing and synthesizing nucleic acids and proteins and for manipulating genes and regulation of gene expression. Genes, however, operate in large part through coding for the production of proteins. Proteins are the major effector molecules of cells, acting as enzymes, receptors, structural components, oxygen carriers, intercellular signals, antibodies, and regulators of gene functions and cell division.

Now the international Human Proteome Organization (HUPO) has launched a global Human Proteome Project (HPP). The foundation for the Project lies in major knowledge bases with systematically organized data about proteins generated from mass spectrometry and from antibody-capture approaches. Scientists in every country can access these open access data repositories to learn about proteins of interest and to initiate their own analyses with these data.

One of the big surprises of the Human Genome Project was the number of protein-coding genes. Originally, the estimate was 50,000 to 100,000 or more; when the nearly complete human genome was published in 2001, the estimate was 35,000. Now a much more reliable estimate is 20,300. How do we perform so many complex developmental, physiological, and disease-related functions with so few genes, in comparison with some other species?

It turns out that, through evolution, individual genes can be transcribed into several different messenger RNA transcripts by a process known as alternative splicing, and those mRNAs can be translated into protein splice variants. Moreover, proteins are covalently modified by addition of sugar, phosphate, acetyl, and other small molecules to the side chains, greatly modifying the functional properties of the initial protein structure. So, characterizing the human proteome (the term for all the proteins) is much more complex even than sequencing the human genome. In addition, while there are exactly two copies of nearly every gene in every nucleated cell of the 230 cell types of the body, the number of copies of a protein may vary enormously over time, in different organs and body fluids, and from protein to protein.

This Project will be important to Global Health since proteins are the molecular targets of most pharmaceuticals and are used in medicine and public health for diagnosis and for vaccines. In biotechnology, many of the most important new products are proteins, including antibodies.

## Organization of the Human Proteome Project

**Figure 1:**
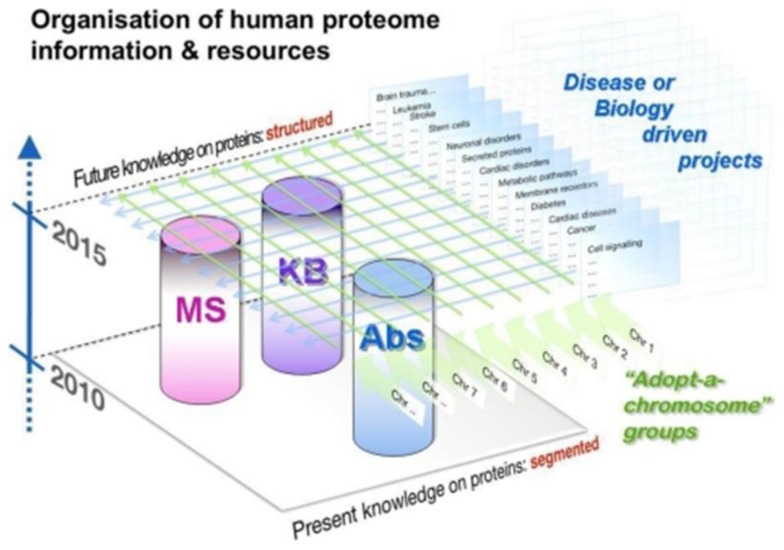
Schema showing the organization of the Human Proteome Project (HPP) with a foundation of Antibody, Mass Spectrometry, and Knowledge Base resource pillars and two major collaborative research initiatives, the chromosome-centric C-HPP and the biology and disease-driven B/D-HPP

Legrain et al.^2^ described the aims and organization of the Human Proteome Project. Scientists from dozens of countries are engaged with specific roles on the HPP Executive Committee, the Senior Scientific Advisory Board, and the operating arms of the HPP (see Figure 1).

As of this time, national and international teams of scientists have initiated studies of proteins coded by genes on individual chromosomes, including teams based in Russia (chromosome 18) and in Iran (chromosome Y), with 23 of the 24 chromosomes now assigned (22 autosomes plus chromosomes X and Y). Other teams of investigators are pursuing a dozen biological and disease-based studies around the theme of the roles of proteins in biological networks from organ, biofluid, model organism, and stem cell proteomes.

The Executive Committee and Principal Investigators Council of the Chromosome-centric C-HPP and the corresponding Executive Committee and Principal Investigators Council of the Biology and Disease-driven B/D-HPP are shown in Figure 1. The Figure also shows the Antibody-based, Mass Spectrometry-based, and Knowledge-based resource pillar committees of the HPP.

A web portal has been established at www.thehpp.org, with a corresponding working group. The website is a good place to learn about and link to data from the HPP. The web portal lists all the members and countries involved in the various components of the HPP. The portal also serves as a knowledge center for educational purposes, highlighting standardization of protocols used in the HPP. Information about the HPP will also appear in the Science Supercourse (www.pitt.edu/~super1 or www.bibalex.org/supercourse/) which is a magnificent resource of PowerPoint lectures created by Dr. Ronald Laporte of the University of Pittsburgh, USA, and now based at the Biblioteca Alexandria in Egypt, led by Dr. Ismail Serageldin.^3^

## Data from the Human Proteome Project

Protein capture datasets based on antibodies and immunohistochemistry of human specimens are presented in the Human Protein Atlas, with information about antibodies in Antibodypedia.^4^ Submission of MS-datasets is standardized through the ProteomeXchange, neXtprot, PRIDE, PeptideAtlas, and GPMdb. ProteomeXchange and PRIDE are based at the European Bioinformatics Institute in Hinxton, England, UK; neXtprot is based at the Swiss Institute for Bioinformatics in Geneva, Switzerland; Peptide Atlas is at the Institute for Systems Biology in Seattle, Washington, USA; and GPMdb, in Alberta, Canada. Peptide Atlas provides uniform reanalysis using the TransProteomicPipeline (TPP), with stringent criteria to minimize false identifications of proteins (1% false discovery rate, at the protein level).

There are now peptide atlases for human and mouse plasma,^5^ the liver, kidneys, urine, and brain. A major advance in proteomics is a targeted approach, identifying and quantitating proteotypic (unique) peptides from each protein of interest, rather than trying to identify as many as possible of the proteins and having the peptides dominated by the most abundant proteins. This method is called Selected Reaction Monitoring (SRM). A PeptideAtlas for SRM Experimental Libraries (PASSEL) has been launched by the Institute for Systems Biology in Seattle, along with spectral libraries and peptide resources that facilitate and democratize SRM studies by groups worldwide.

The C-HPP and B/D-HPP programs collaborate in building the parts list of proteins corresponding to the 20,300 protein-coding genes, plus post-translational modifications (PTMs), splice variants (see above), and polymorphic mutations or single nucleotide polymorphisms (SNPs). The C-HPP^6^–^7^ aims to reveal co-expressed proteins of co-located genes; the B/D-HPP is defining a framework of protein networks and interactions. HPP investigators will analyze unusual specimens (nasal epithelium, placenta, fetus, brain regions) to detect “missing proteins”, use ultrasensitive methods for low-abundance proteins, identify and differentiate protein families, splice variants, and PTMs, and deduce the functional features of these proteins and their isoforms.

## Deliverables

The Human Proteome Project will generate (a) structured information about human proteins (the protein parts list), including both the approximately 13,000 gene products for which some protein information is already known and the 7000 proteins for which there is no evidence yet at the protein level; and (b) reagents and tools for additional protein studies. The investigators will devise metrics to describe annually the extent of progress on identifying and characterizing the human proteome.

Within 3–5 years we expect to have SRMbased spectral libraries for multiple proteotypic peptides for at least one protein product of each of the 20,300 protein-coding genes, with an SRM Atlas and stably labeled peptide standards. We expect to have an expanded Human Protein Atlas with polyclonal antibodies to characterize the tissue expression and subcellular localization of more than 12,000 gene products; in fact, Uhlen and colleagues have announced findings for 13,985 proteins at the 2012 HUPO World Congress of Proteomics and have published detailed findings for the proteins coded for on chromosome 21.^4^ We will stimulate cross-comparisons of tissue expression by antibody and mass spectrometry methods, and datasets will be captured through ProteomeXchange in standardized formats, as noted above.

Over a period of up to 10 years we will extend SRM analyses and knowledge bases to splice variants and post-translational modifications of proteins. We will have renewable protein capture methods and reagents (monoclonal antibodies; perhaps aptamers) for characterization of protein tissue expression and localization, including responses to physiological and pathological perturbations. We will have a greatly expanded set of data repositories.

We expect that the HPP will enable and encourage other scientists, beyond the basic research community, to use or target proteins for diagnosis, prognosis, prevention, therapy, and cure of diseases. Such applications will improve human health worldwide.

## Conclusion

Completion of the Human Proteome Project will enhance understanding of human biology at all levels, from individual cells to populations, and will lay a foundation for diagnostic, prognostic, therapeutic, and preventive applications. The Human Proteome Organization urges each national scientific community and its research funding agencies to identify their preferred pathways to participate in aspects of this highly promising Project.

## Figures and Tables

**Figure 2:**
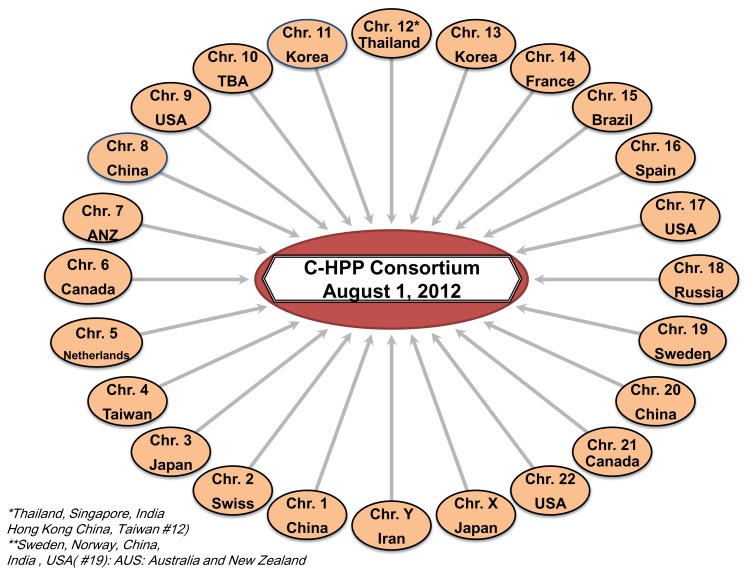
The research teams responsible for each of the chromosomes in the chromosomecentric C-HPP, by lead nation.
